# Neurophysiological correlates of passive movements are speed- and type-dependent

**DOI:** 10.1007/s00421-026-06172-2

**Published:** 2026-03-02

**Authors:** M. P. Veldman, J. Z. Kwant, J. Lommerse, M. Feenstra, C. J. C. Lamoth, A. H. M. Volkers, H. Drenth, S. Zuidema, I. Bautmans, H. Hobbelen

**Affiliations:** 1 Department of Human Movement Sciences, University of Groningen, University Medical Center Groningen, Antonius Deusinglaan 1, FA23, 9713AV Groningen, The Netherlands; 2 Allied Health Care and Nursing, Research Group Healthy Ageing, Hanze University of Applied Sciences Groningen, Groningen, The Netherlands; 3 ZuidOostZorg, Center for Elderly Care, Drachten, The Netherlands; 4 Department of Primary and Long-Term Care, University Medical Center Groningen, University of Groningen, Groningen, The Netherlands; 5 Frailty & Resilience in Ageing Research Unit, Vitality Research Group, Vrije Universiteit Brussel, Brussels, Belgium; 6 Gerontology Department, Vrije Universiteit Brussel, Brussels, Belgium; 7 Geriatrics Department, Universitair Ziekenhuis Brussel, Brussels, Belgium; 8 Geriatric Physiotherapy Department, SOMT University of Physiotherapy, Amersfoort, The Netherlands; 9 Move-in-Age International Joint Research Group, Vrije Universiteit Brussel, Brussels, Belgium

**Keywords:** Passive movement, Electroencephalography, Aging, Inhibition, Corticomuscular coherence

## Abstract

**Purpose:**

The supraspinal involvement in the control of passive movements remains elusive. Previous studies provided electromyographic evidence for speed and –type dependent changes in muscle activity. Based on mechanoreceptor properties, their age-related changes and the somatotopically organized connections between sensory and motor systems, this study aimed to provide electrophysiological evidence for the involvement of frontal cortex inhibition and corticomotor interactions in the control of passive movements.

**Methods:**

Twenty healthy younger and older adults performed passive elbow flexion and extension movements at three metronome-based speeds (20, 60, and 100 beats per minute) in continuous and discontinuous fashion. The continuous condition included movements with no inter-movement pauses and the discontinuous conditions with pauses between flexion–extension transitions. The order of movement speeds increased progressively to prevent carryover effects in hypothesized resistance to passive movements from higher to lower speeds. In all conditions, electro-encephalographic and electromyographic data were acquired. Alpha power and beta corticomuscular coherence were used to quantify frontal cortex inhibition and brain-muscle connectivity, respectively.

**Results:**

Frontal cortex inhibition decreased (p = 0.036) and brain-muscle connectivity increased (p < 0.001) with increasing movement speeds. In addition, frontal cortex inhibition was 17% higher in the discontinuous condition as compared to the continuous condition (p = 0.005) while corticomuscular coherence was 25.9% higher in the continuous vs. the discontinuous condition (p < 0.001). These effects were independent of age.

**Conclusion:**

Frontal cortex inhibition and brain-muscle interactions depend on passive movement speed and -type. The current data may provide insights into the processes underlying pathological muscle tone during passive movements.

## Introduction

Passive movements influences human functioning at various levels. At the muscle architecture level, muscle fibers are combined to whole muscles by connective tissue that passively moves along with contractions. The structural organization of connective tissue therefore substantially influences the magnitude of force that muscles exert during contractions (Enoka 2015). Changes in muscle length during contractions—induced by both active and passive movements – are detected by various mechanoreceptors, that in turn affect bodily functions. Stretch-sensitive type III mechanoreceptors play a role in increasing ventilatory and cardiovascular responses at the organ level [for a review, see (Trinity and Richardson 2019)] as well at as the muscle level. Within muscles, muscle spindles detect (the rate of) change in muscle fiber length and send afferent volleys to the spinal cord via type Ia and II afferent nerves that may trigger spinal reflexes (Enoka 2015). These sensory afferents ascend to the primary sensory cortex and form direct, somatotopically organized connections with the primary motor cortex [for a review, see (Veldman et al. 2014)]. As such, passive movements uniquely allow the isolation of afferent sensory feedback and examination of afferent-driven cortical responses, free from the confounding influence of voluntary motor control.

Passive movements indeed elicit cortical activity. For example, conjunction analyses of functional imaging data revealed overlap in activity in sensorimotor and cerebellar areas when healthy young individuals performed active and passive pedaling movements [e.g., (Jaeger et al. 2014)]. Since mechanoreceptors detect rates of change, and their inputs reach supraspinal structures, this provides neuroanatomical and neurophysiological support that cortical activity varies with different passive movement speeds. Moreover, since muscle spindle sensitivity decreases with advancing age (Lord et al. 2018), the effect of passive movements on cortical activity may also change with age. Indirect support for this hypothesis comes from a series of studies by Marinelli and colleagues, suggesting an age- and movement-speed-dependent association between passive movements and cortical activity. They observed involuntary increases in electromyographic (EMG) activity in healthy individuals, with these increases being more pronounced in older age groups and during fast, continuous movements (Marinelli et al. 2017, 2022). Notably, this passive-movement-related phenomenon was clinically relevant since these findings were amplified in individuals with cognitive decline, offering insights into the mechanisms underlying paratonia, a dementia-related motor symptom that is characterized as involuntary resistance to passive movements. A prominent hypothesis postulates that pathological responses to passive movements may originate from frontal cortex dysfunction (Beversdorf and Heilman 1998; Drenth et al. 2020), which is in line with data showing that motor output is under inhibitory control of the prefrontal cortex (Aron et al. 2004) and that passive movements elicit activity in frontal areas (Jaeger et al. 2014).

Direct neurophysiological evidence for this hypothesis, however, is missing. In this study, we use electroencephalography (EEG), that measures oscillatory brain activity at the scalp with a high temporal resolution, to quantify frontal cortex inhibition and brain-muscle connectivity. Specifically, previous research showed that oscillations in the alpha frequency range (8–12 Hz) reflect inhibitory processes (Klimesch et al. 2007). Moreover, because oscillations at the beta frequency range have been shown to be implicated in motor processes (Pfurtscheller and Lopes da Silva 1999; van Wijk et al. 2012), the coherence between EEG signals from electrodes located over the motor cortex and EMG signals, i.e., corticomuscular connectivity, reflects brain-muscle interactions and is sensitive to age-related changes [e.g., (Spedden et al. 2019)].

The present study aims to provide direct electrophysiological evidence for the hypothesis that neurophysiological responses to passive movements are age- and speed-dependent. Based on properties of mechanoreceptors and previous findings in the context of paratonia (Marinelli et al. 2017, 2022), we hypothesized that (1) frontal cortex inhibition decreases with increasing age and movement speed and is lower in continuous vs. discontinuous movements based on elevated EMG-levels during such movements in a previous study (Marinelli et al. 2017). Because other data show that higher muscle tone in people with spasticity occurs in absence of increases in fusimotor drive from the spinal cord suggestive of disinhibition [for a reviews, see (Sheean 2002; Nordin et al. 2017)], we also hypothesized that (2) brain-muscle connectivity increases with age and movement speed, and that the magnitude of such connectivity is higher in continuous vs. discontinuous movements.

## Methods

### Participants

Healthy young (n = 20, 22.5 ± 2.31 y, 9 females, two left-handed) and older (n = 20, 72.7 ± 5.73 y, 6 females, none left-handed) adults participated in this study. The sample size was based on a priori power analysis for repeated measures analysis of variance (ANOVA; effect size: 0.25; power: 0.8). Participants were recruited from Groningen and the surrounding area by local flyers and mouth-to-mouth advertising. They were included if they were free of neurological disorders or other physical and psychological disorders and did not take drugs that affect nerve conduction velocity or cognitive processes, such as antidepressants, antipsychotics or sleep medications. Participants provided written informed consent before participating in a protocol that was approved by the Medical Ethical Review Committee (NL81562.042.22) of the University Medical Center Groningen and was conducted according to the Declaration of Helsinki (2013).

### Experimental design

This study adopted a cross-sectional design based on previous work (Marinelli et al. 2017) that was extended with synchronous EEG and EMG recordings. Baseline measures included anthropometrics, handedness [Edinburgh Handedness Inventory, (Oldfield 1971)], physical [Timed Up and GO, (Podsiadlo and Richardson 1991)] and cognitive [Montreal Cognitive Assessment, (Nasreddine et al. 2005)] function. In addition, the Paratonia Assessment Instrument was performed to subjectively assess the presence of paratonia by interpreting the extent of resistance during passive movements (Hobbelen et al. 2008).

### Experimental protocol

**Fig. 1:**
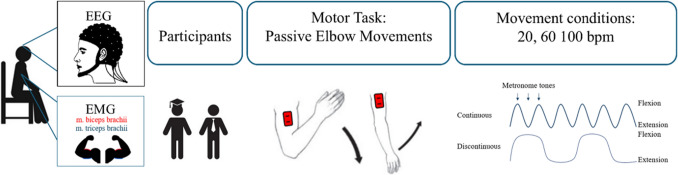
Overview of the experimental design. In healthy younger (n = 20) and older (n = 20) individuals, continuous and discontinuous passive movements were performed at three different speeds (20, 40 and 60 bpm) while 64-channelelectroencephalography (EEG) and biceps and triceps surface electromyography (EMG) data were acquired. bpm, beats per minute

The experimental protocol started with a seated control task after which a series of arm movements with the right arm followed while brain (EEG) and muscle (EMG) activity was recorded. During the experiment, participants sat on a chair with their knees flexed at a ninety-degree angle and their feet flat on the ground. Participants performed passive elbow flexion and extension movements with the dominant right arm at three different speeds, i.e., 20, 60 and 100 beats per minute (Fig. 1). The participants were instructed to relax their arm during the passive arm movements while the researcher moved the arm through its full range of motion thirty times (i.e., thirty flexion and thirty extension movements). This research focused on upper-limb movements allowed for a rigorous and controlled investigation of the core neural mechanisms under study and enabled direct comparison with previous studies (Marinelli et al. 2017) without substantially increasing experimental complexity and session duration associated with including lower-limb movements – factors that reduce feasibility in clinical populations. At all three speeds, the passive movement conditions were performed in a continuous (i.e., one beat of the metronome between maximal flexion and maximal extension) and discontinuous (i.e., one beat of the metronome between maximal flexion and maximal extension and one beat of the metronome of pause between the end of a flexion/extension movement and the start of the next extension/flexion movement) fashion, resulting in a total of six conditions. All arm movements were performed on the rhythm of a metronome (Tempo Lite, Frozen Ape Pte. Ltd.), which was exclusively audible to the researcher through earphones. The order of the movement type was pseudorandomized so that half of the participants started with the continuous movements and the other half with the discontinuous movements. The order of movement speed was not randomized and were performed with increasing movement speed as to prevent that hypothesized resistance to passive movements during higher movement speeds influenced conditions with slower movement speeds. Breaks were allowed depending on the participant’s need for rest. Figure 1 provides a schematic overview of the experimental design.

### Electrophysiological data acquisition and analysis

#### Data acquisition

Muscle activity from the right M. biceps brachii and M. triceps brachii were recorded using surface wireless EMG sensors (Trigno Avanti, Delsys, Natick, MA, USA; 27 × 37 × 13 mm). The EMG sensors were placed over the right biceps and triceps brachii muscle bellies in the direction of the underlying muscle fibers guided by palpation during contraction (see Fig. 1), which were determined by palpating the muscle during voluntary muscle contraction. EMG data were acquired with bipolar recordings at a sampling frequency of 1926 Hz.

Electroencephalography (EEG) data were continuously acquired with a 64-channel EEG system with Ag–AgCl electrodes placed on the scalp according to the international 10–20 system (TMSi SAGA64 + , Oldenzaal, the Netherlands). The EEG data were acquired at 2048 Hz with an average reference. The impedance was kept below 10 kΩ. To minimize noise in the EEG signals, participants were asked to not speak, to avoid head movements, to relax the jaw and neck muscles and to minimize eye blinking during the measurements. A hard-wired trigger (0–5 V ramp-up TTL trigger) ensured the synchronous acquisition of EEG and EMG data.

#### Data preprocessing

The EEG data were pre-processed in MATLAB R2022b (MathWorks), using scripts based on FieldTrip (Oostenveld et al. 2011). Because the frequencies ranges of interest in this study were alpha (8–12 Hz) and beta (13–30 Hz), the data were filtered with a 3 Hz high-pass filter (6th order Butterworth) and a 70 Hz low-pass filter (6th order Butterworth). This way, we removed low-frequency drifts and high-frequency noise (for example from EMG) while sufficient data was retained for adequate rejection of residual artefacts with independent component analyses (see below). To remove residual electromagnetic line noise, a notch filter with a frequency of 50 Hz and its second and third harmonic (100 and 150 Hz) was also applied. After resampling the data to 1024 Hz with a piecewise cubic hermite interpolating polynomial using Fieldtrip’s ft_resampledata function, EEG channels were visually inspected and empty channels or channels with substantial artifacts were removed. Next, an independent component analysis was performed to remove artifacts caused by eye movements and muscle contractions based on time series and topographical distributions of power. This process was repeated for each condition and consistently performed by the same researcher (AHMV). On average, three components per trial were removed. The preprocessing pipeline concluded with referencing the data to an average reference.

The EMG data was pre-processed in MATLAB R2022b (MathWorks). First, the EMG data were restructured in the FieldTrip format to enable the preprocessing of the EMG data using similar procedures as for the EEG data. The EMG data were bandpass filtered using a 4rd order Butterworth filter between 4 and 100 Hz to remove low-frequency drifts and high-frequency noise while retaining the frequencies of interest (13 – 30 Hz). Additionally, electromagnetic line noise was removed with a 4rd order Butterworth notch filter of 50 Hz and its second and third harmonic (100 and 150 Hz). The EMG data were full-wave rectified using an absolute Hilbert transform and resampled to 1024 Hz, again with a piecewise cubic hermite interpolating polynomial using Fieldtrip’s ft_resampledata function.

#### Data analysis

The preprocessed EEG time series were epoched into non-overlapping 1-s-long epochs before being transformed to the frequency domain using a multitaper Fast Fourier Transformation with a Hanning window leading to a 1 Hz frequence resolution. Power in the alpha frequency range (8–12 Hz) was averaged for five frontal electrodes [F3, Fz, F4, FC3 and FC4; (Gompf et al. 2017)].

To quantify brain-muscle interactions, EEG and EMG data were concatenated and epoched in non-overlapping 1-s-long segments before being transformed to the frequency domain between 1 and 40 Hz using a multitaper Fast Fourier Transformation method with 5 Hz smoothing. Subsequently, corticomuscular coherence was calculated using the auto- and cross spectra (Halliday et al. 1995). Then, beta-range (13 – 30 Hz) corticomuscular coherence was estimated between the pre-processed EEG timeseries in the electrode positioned over the upper arm region of the primary motor cortex contralateral to the right arm, represented by the C3, and the pre-processed EMG timeseries recorded from the biceps and triceps muscles. For each condition, the level of corticomuscular coherence was quantified as the area under the curve between the corticomuscular coherence estimates and significance line of the amplitude spectrum in the frequency domain (Amjad et al. 1997).

### Statistical analysis

Statistical analyses were performed in SPSS (Version 26, IBM, Chicago, IL, USA). The Shapiro–Wilk test revealed that all data were normally distributed. Descriptive statistics (in means and standard deviations) were presented for baseline age, physical and cognitive performance, and handedness by age group. To examine whether age, movement speed and movement type impacted alpha power data and beta corticomuscular coherence, data were analysed with repeated measures ANOVA for each hypothesis. Movement speed (20, 60 and 100 bpm) and type of movement (continuous and discontinuous) were included as within-subject factors and age (healthy younger and older) was included as between-subjects factor. The significance level of the Box’s M test was set at p < 0.001, as this test is considered highly sensitive (Jiamwattanapong and Ingadapa 2021). When the assumption of sphericity was violated as evidenced by the Mauchly’s test, a Greenhouse–Geisser correction of degrees of freedom was used. Partial eta square (η_p_^2^) was used as a measure of effect size where ≥ 0.2, ≥ 0.5 and ≥ 0.8 were considered as small, medium and large effects, respectively (Cohen 2013). For all analyses the level of significance was set at p < 0.05.

## Results

Seventeen older and twenty younger individuals completed this study. Three of the included older adults were excluded from further participation because they scored < 26 on the Montreal cognitive assessment. Subjective paratonia was observed in two older adults and not in younger adults. Table 1 provides an overview of the baseline measures.

**Table 1:** Descriptive statistics

	Older adults (n = 20)	Younger adults (n = 20)
Age in years (mean ± SD)	72.7 ± 5.7	22.5 ± 2.3
Sex (M/F)	14/6	11/9
MoCA^1^ (mean ± SD)	26.1 ± 2.6	27.6 ± 1.7
TUG^2^ (s) in seconds (mean ± SD)	7.87 ± 1.0	6.8 ± 1.4
EHI^3^	96.7 ± 5.9	66.2 ± 54.3

*MoCa* montreal cognitive assessment, *TUG* timed up and go, *EHI* Edinburgh handedness inventory

1Higher scores indicate better performance.

2Higer scores indicate worse performance.

3Higher scores indicate stronger right-handedness.

**Fig. 2:**
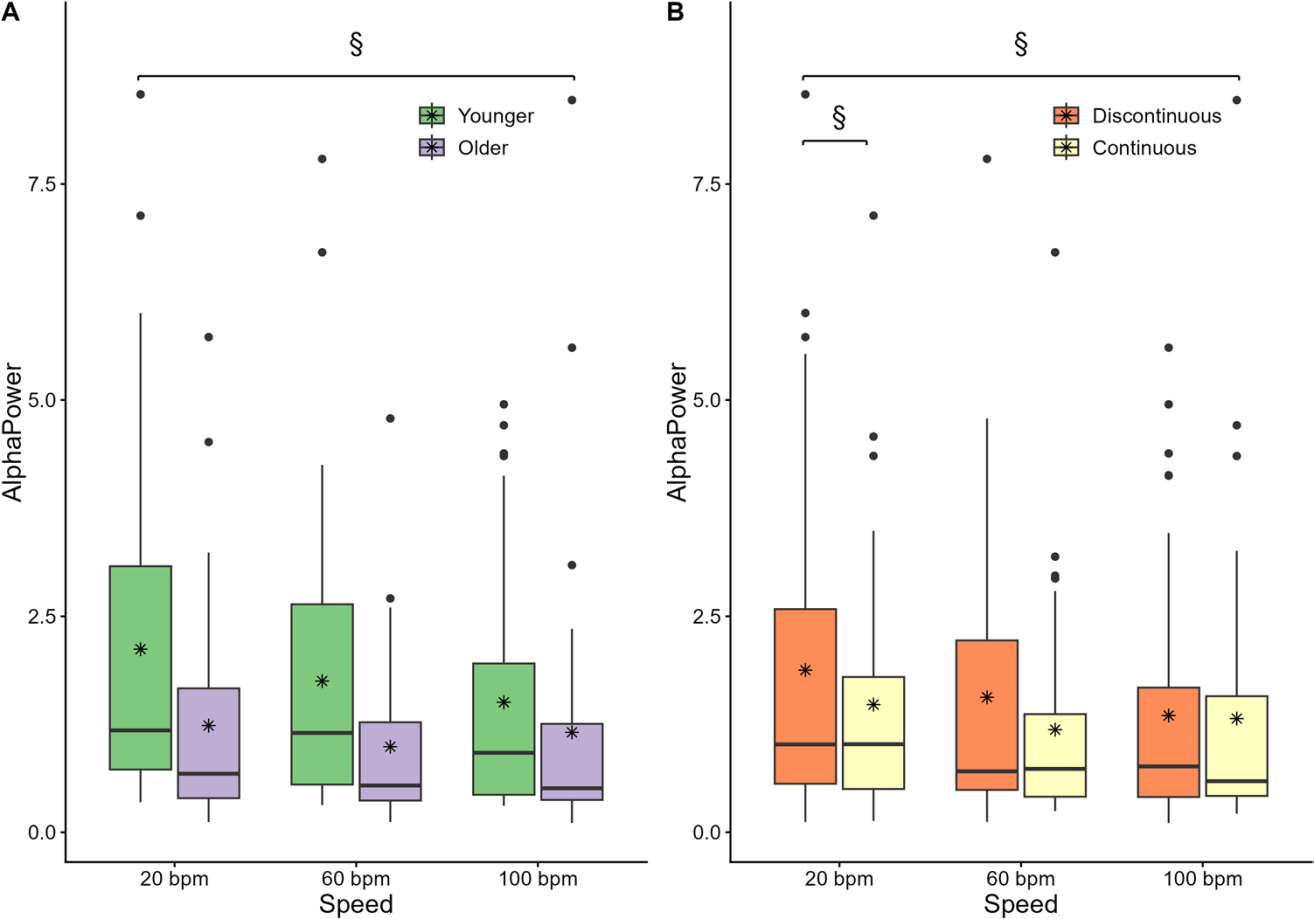
Effects of age, movement speed and movement type on alpha power. The panels display the effects of speed (panels A-B) and movement type (panel B) on alpha power. The horizontal lines and asterisks within the boxes represent the median and mean for each group/condition, respectively. Bpm, beats per minute; §, main effects of speed and condition with p < 0.05

Alpha power decreased with increasing movement speeds (F_(1.558, 52.978)_ = 3.91, p = 0.036, η_p_^2^ = 0.103; Fig. 2A, B) and was 17% higher in discontinuous as compared to continuous movements (F_(1, 34)_ = 8.85, p = 0.005, η_p_^2^ = 0.206; Fig. 2B). There was no effect of age (F_(1, 34)_ = 1.262, p = 0.269, η_p_^2^ = 0.036) and none of the interaction effects reached significance.

**Fig. 3:**
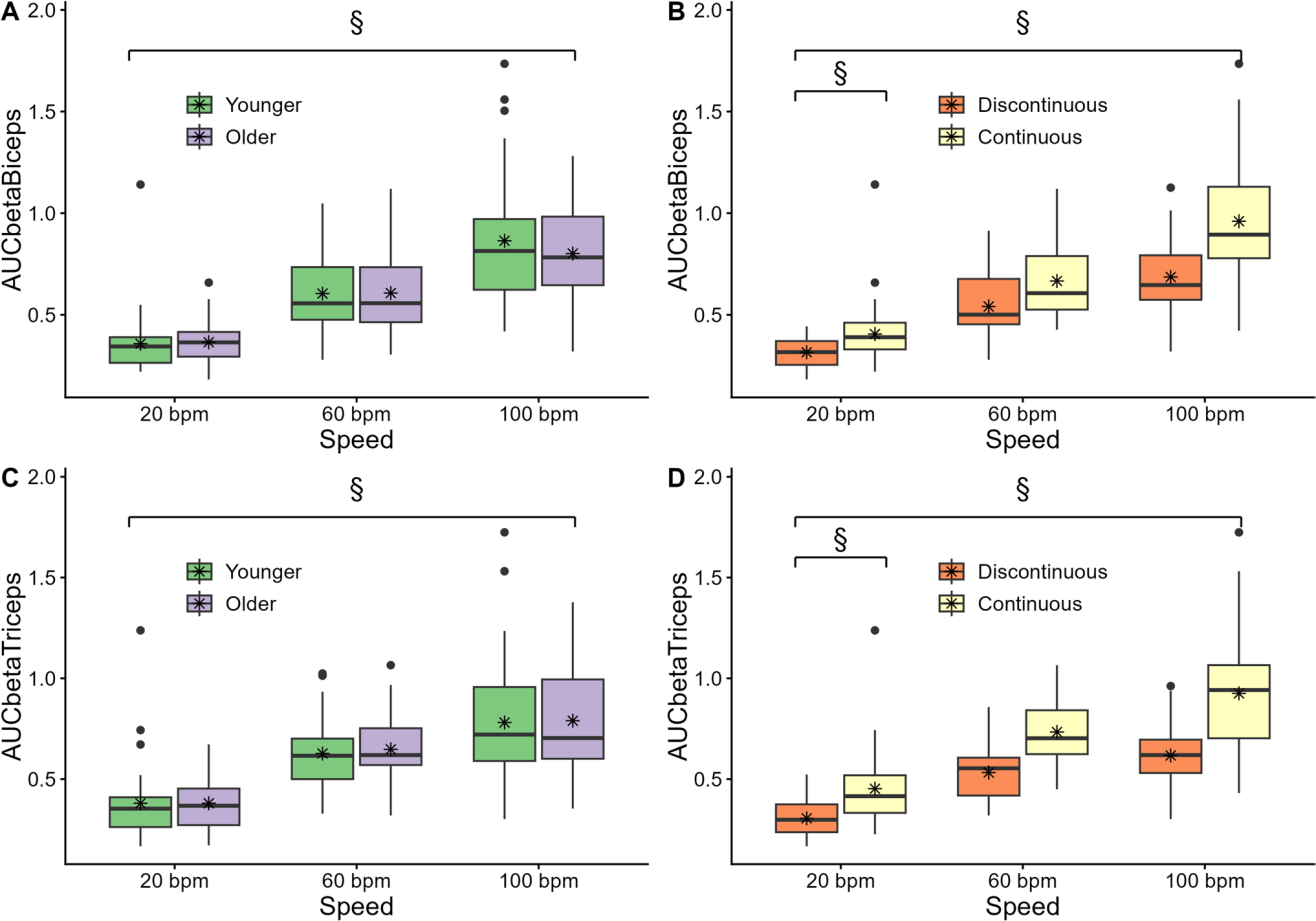
Effects of age, movement speed and movement type on corticomuscular coherence. The panels display the effects of speed (panels A-D) and movement type (panels B and D) on corticomuscular coherence between the primary motor cortex and the biceps muscle (panels A-B) and triceps muscle (panels C-D). The horizontal lines and asterisks within the boxes represent the median and mean for each group/condition, respectively. Bpm, beats per minute. §, main effects of speed and condition with p < 0.05

Corticomuscular coherence between the primary motor cortex and the biceps muscle increased with increasing movement speed (F_(2, 26)_ = 108.04, p < 0.001, η_p_^2^ = 0.820; Fig. 3A, B). Additionally, the continuous condition showed 25.9% higher corticomuscular coherence as compared to the discontinuous condition (F_(1, 27)_ = 27.08, p < 0.001, η_p_^2^ = 0.599; Fig. 3B). Similarly, corticomuscular coherence between the primary motor cortex and the triceps muscle increased with increasing movement speed (F_(1.654, 44.663)_ = 77.26, p < 0.001, η_p_^2^ = 0.741; Fig. 3C, D) and was 32.1% higher in continuous vs. discontinuous movements (F_(1, 27)_ = 40.38, p < 0.001, η_p_^2^ = 0.599; Fig. 3D). There were no effects of age (Fig. 3A–C) or interaction effects.

## Discussion

This study examined the effects of age, movement speed and movement type on frontal cortex inhibition and brain-muscle connectivity during passive elbow flexion and extension movements. In line with the hypotheses, frontal cortex inhibition, reflected by alpha power, decreased and brain-muscle connectivity, reflected by corticomuscular coherence, increased with increasing movement speeds. In addition, discontinuous movements were associated with higher inhibition and lower brain-muscle connectivity as compared to continuous movements. In contrast with our hypothesis, however, age did not affect frontal cortex inhibition and brain-muscle connectivity. The present study altogether reinforces the hypothesis of supraspinal involvement in the control of passive movements as indicated by movement speed and -type-dependent modulation of frontal cortex inhibition and brain-muscle connectivity.

### Movement speed

The decrease in frontal cortex inhibition and increase in brain-muscle connectivity with increasing movement speeds are in line with our hypothesis and consistent with other studies showing decreased alpha power over sensorimotor regions with increasing speeds in a walking task (Nordin et al. 2020). These data suggest that the interplay between sensory inputs and motor outputs is important during the control of passive movements. It is likely that input from muscle spindles that detect the (rate of) change in muscle fiber length (Enoka 2015), and reaches the primary and motor cortex via somatosensory afferents and somatotopic connections between the primary sensory and motor cortices (Veldman et al. 2014), played a role in the current findings. Imaging data suggest that the overlap in neural activation between voluntary and passive movements exceeds the primary sensorimotor regions and extends to frontal regions (Dobkin et al. 2004; Jaeger et al. 2014). Interestingly, peak activations during passive movements are generally weaker as compared to voluntary movements, except for activations in the secondary motor cortex (Dobkin et al. 2004), that has been suggested to be a mediating structure between sensory and motor areas based on animal data (Barthas and Kwan 2017). Such interactions between sensory and motor systems may also explain the decreased inhibition in the frontal cortex. Specifically, decreased inhibition allows for increased responsiveness to incoming sensory information associated with faster movements (Mathewson et al. 2011; Klimesch 2012), which may have triggered the increases in connectivity [(Jones 1983; Terao et al. 1999), for a review see (Veldman et al. 2014)]. This suggestion is further supported by data showing event-related desynchronization in the beta range (Alegre et al. 2002), which is differently modulated depending of the movement phase, i.e., preparation, execution, termination [e.g., (Pfurtscheller and Lopes da Silva 1999; Heinrichs-Graham et al. 2017)]. In summary, as passive arm movement speed increases, frontal cortex inhibition decreases and brain-muscle connectivity increases, possibly via a complex interplay between sensory afferents and motor efferents.

### Continuous vs. discontinuous movements

The higher frontal cortex inhibition and reduced brain-muscle connectivity during discontinuous, as compared to continuous, movements may reflect the increased need to control motor output in discontinuous movements. The nature of the discontinuous movements requires repeated stopping and starting of an ongoing process. In this context, muscle spindles, which respond to the rate of change of muscle fiber length and subsequently may result in reflexive contraction of the muscle via the muscle spindle reflex arc (Macefield and Knellwolf 2018), are arguably more active in discontinuous movements in comparison with continuous movements. Since participants were instructed to relax their muscles during the passive movements, we speculate that the increased alpha power in the frontal cortex could reflect active inhibition of this muscle spindle reflex arc that may have been necessary to prevent the reflexive contraction that is required for allowing passive movements.

The interpretation of more active inhibition during discontinuous movements would logically lead to the prediction that brain-muscle connectivity is lower in discontinuous as compared to continuous movements. The present study supports this prediction as the magnitude of brain-muscle connectivity was lower in discontinuous versus continuous movements. These findings are consistent with previous data indicating modulations of brain-muscle connectivity following passive movements in a walking neurorehabilitation context (Artoni et al. 2023), suggesting an overlap between neurophysiological underpinnings of active and passive movements. Moreover, because beta-range corticomuscular coherence has been implicated in the coordination of activity between different muscles (Reyes et al. 2017), the higher corticomuscular coherence in the continuous movement condition might reflect a higher degree of coordination between biceps and triceps activity in this condition. This interpretation is further supported by the absence of differences between the M. biceps and M. triceps brachii on brain-muscle connectivity. Altogether, the current and previous data indicate that supraspinal structures are more involved in continuous passive movements as compared to discontinuous movements.

### Age

Contrary to the hypothesis, the modulation of frontal cortex inhibition and brain-muscle connectivity was independent of age. Previous studies revealed inconsistent age-related modulations in beta-range brain-muscle connectivity, with some showing age-related increases (Johnson and Shinohara 2012; Kamp et al. 2013) while others reported age-related decreases in beta-range CMC (Bayram et al. 2015). Our hypothesis that such age-related differences would be present during passive movements was based on the deterioration of muscle spindle sensitivity with advancing age (Lord et al. 2018) and inspired by previous data showing age-related increases in the presence of paratonia (Marinelli et al. 2017, 2022). Participants included in the final analyses, however, did not show signs of paratonia as assessed by the subjective paratonia assessment instrument (Hobbelen et al. 2008). We speculate that the interactions between sensory and motor systems are relatively robust against slight reductions in sensory function and that more advanced sensory deteriorations are required to detect age-related changes at the cortical level. In this context, it is noteworthy that of the three participants who were excluded from the analyses because they scored < 26 on the the MoCA, two showed slight to moderate signs of paratonia based on the subjective paratonia assessment instrument. If the current assumption that paratonia is mediated by motor cortex disinhibition as a result of frontal cortex dysfunction in people with dementia is true, the absence of age-related effects in the present sample is not surprising.

### Clinical implications

The control of passive movements is affected in a number of neurodegenerative pathologies. In Parkinson’s disease, the unintentional display of heightened muscle tone in response to passive movements, hypertonia and “lead-pipe” rigidity, can only partly be attributed to short- and long-latency stretch reflexes (Xia et al. 2011; Kwon et al. 2014), which suggest a cortical origin of these symptoms. Indeed, in people with spasticity, heightened muscle tone is observed in the absence of increased fusimotor drive from the spinal cord suggestive of cortical disinhibition [for a review, see (Nordin et al. 2017)]. The current study design was based on previous studies investigating paratonia, the unintentional resistance to passive movements in people with dementia (Marinelli et al. 2017, 2022). Such impaired motor control has a severe impact on daily life, not only in performing activities of daily living but also in the care of individuals who can no longer actively manage these tasks themselves. Moreover, our data are consistent with electrophysiological data in people with paratonia that display more muscle activity at higher movement speeds and during continuous movements as compared to discontinuous movements. Furthermore, the current findings provide initial insights and support for the hypothesis that frontal cortex disinhibition play a role in the increased resistance to passive movements (Drenth et al. 2020). Future studies in people with dementia that show signs of paratonia can corroborate the hypothesis that frontal cortex dysfunction cause diminished control over passive movements. Together, these studies may pave the way for the development of targeted interventions for the treatment of pathological muscle tone. For example, if our hypothesis that frontal cortex disinhibition underly paratonia in individuals with dementia as reflected by higher corticomuscular connectivity during passive movements, our approach could be applied to other neurological populations with spasticity-like symptoms, such as upper motor neuron syndrome. Collectively, these future studies would be able to identify shared neurophysiological mechanisms underlying pathological muscle tone. If so, interventions that are effective in some disorders may then also be effective other neurological conditions with symptoms caused by similar neurophysiological mechanisms. Such interventions may include, but are not limited to, neuromodulatory electrical or magnetic stimulation techniques (e.g., transcranial electrical direct current stimulation or magnetic theta-burst stimulation) targeting the magnitude of frontal cortex inhibition, thereby restoring inhibitory balance and prevent disinhibition of the motor cortex and its associated pathological muscle tone.

### Limitations

While the experimental design systematically evaluated the effect of passive movement speed and type and the passive movements were induced by trained experimenters, the personal approach may have resulted in slight variations in movement speed and amplitude. It is possible that these variations triggered involuntary movements or resistance to movements and consequently affected our neurophysiological outcome measures. Moreover, because we focused on the cortical and corticomotor correlates of passive movements, we opted to not control for the amplitude of the acquired EMG signals. Although alpha power is likely insensitive to the magnitude of the EMG signals and corticomuscular coherence is a normalized outcome measure, it would be interesting for future studies to evaluate whether alpha power in the frontal cortex and beta corticomuscular coherence are modulated with varying EMG levels. Lastly, as depicted in Figs. 2 and 3, the EEG-derived outcome measures, alpha power and beta brain-muscle interactions, displayed considerable variation, which may have reduced the statistical power and obscured age-related differences.

## Conclusion

This study evaluated whether the neurophysiological correlates of passive movements depend on age, movement speed and type. The results revealed that, independent of age, frontal cortex inhibition decreased and brain-muscle connectivity increased with increasing movement speeds and that continuous, as compared to discontinuous, movements were associated with lower frontal cortex inhibition and higher brain-muscle connectivity. While future studies in patient populations are necessary to corroborate the prevalent hypothesis that frontal cortex function is involved in conditions that involve pathological muscle tones, the current data provide direct electrophysiological evidence that neurophysiological responses to passive movements are speed- and type-dependent.

## Data Availability

Due to data protection, the datasets collected and analyzed during this study are not openly available. However, data supporting the findings of this study are available from the corresponding author upon reasonable request.

## Abbreviations

ANOVA: Analysis of variance
EEG: Electroencephalography
EMG: Electromyography
